# Health Impact and Social Value of Interventions, Services, and Policies: A Methodological Discussion of Health Impact Assessment and Social Return on Investment Methodologies

**DOI:** 10.3389/fpubh.2020.00049

**Published:** 2020-02-27

**Authors:** Kathryn Ashton, Lee Parry-Williams, Mariana Dyakova, Liz Green

**Affiliations:** ^1^Policy and International Health, WHO Collaborating Centre on Investment for Health and Well-being and the Wales Health Impact Support Unit, Public Health Wales NHS Trust, Cardiff, United Kingdom; ^2^WHO Collaborating Centre for Healthy Urban Environments, University of West of England, Bristol, United Kingdom

**Keywords:** health impact, social value, HIA, SROI, health economics, wider determinants

## Abstract

**Introduction:** Assessing the positive and negative impact of policies, services and interventions on health and well-being is of great importance to public health. Health Impact Assessment (HIA) and Social Return on Investment (SROI) are established methodologies which assess potential effects on health and well-being, including social, economic and environmental factors, indicating synergies, and cross-over in their approach. Within this paper, we explore how HIA and SROI could complement each other to capture and account for the impact and social value of an assessed intervention or policy.

**Methods:** A scoping review of academic and gray literature was undertaken to identify case studies published between January 1996 and April 2019 where HIA and SROI methodologies have been used to complement each other previously. Semi-structured interviews were carried out with nine international experts from a range of regulatory and legislative contexts to gain a deeper understanding of past experiences and expertise of both HIA and SROI. A thematic analysis was undertaken on the data collected.

**Results:** The scoping review identified two published reports on scenarios where HIA and SROI have both been used to assess the same intervention. Results from the interviews suggest that both methods have strengths as standalone methodologies. HIAs were noted to be well-structured in their approach, assessing health and well-being in its broadest context. SROI was noted to add value by monetizing social value, as well as capturing the social and environmental impact. Similarities of the two methods was suggested as their strong emphasis on stakeholder engagement and common shared principles. When questioned how the two methods could complement each other in practice, our results indicate the benefits of using HIA as an initial exploration of impact, potentially using SROI subsequently to monetarize social value.

**Conclusion:** HIA and SROI have many synergies in their approaches. This research suggests potential benefits when used in tandem, or combining the methods to assess impact and account for social value. Further research is needed to understand the implications of this in practice, and to understand how the results of the two methods could be used by decision-makers.

## Introduction

The world is continuously changing, now faster than ever, creating a global transformation of unprecedented scale, scope, and complexity. Social, environmental and economic imbalance threaten all, especially the most vulnerable. Globalization processes have direct and indirect impacts on human health and well-being, and on our planet, pushing the sustainability of systems to their limits. Innovative, integrated and sustainable solutions are urgently needed to ensure peace and prosperity, and the well-being of people and our planet. This requires commitment and comprehensive action, involving stakeholders from all governance levels, across the public and private sectors, the academia and civil society.

Developing and implementing new, as well as reforming old, policies, planning initiatives, services, and interventions can have both positive and negative impacts on the health and well-being of individuals, communities, and populations, as well as on their surrounding physical and socio-economic environment (1, 2). Assessing the possible multiple impacts and measuring the related value using well-established robust methodologies can enable decision-makers to take evidence-informed action for the benefit of those directly or indirectly affected (3), or mitigate for any potential unintended negative impacts.

Health Impact Assessment (HIA) and Social Return on Investment (SROI) are two established methods, which are used to assess the range of health and wider outcomes and impacts of different public policies, services, and interventions. Both methods apply an inter-sectoral cross-cutting approach, which allows impact assessment to be performed by involving society and key stakeholders (those directly affected by or having an interest in the assessed policy, service or intervention) in a participatory way. In addition, both HIA and SROI aim to capture the holistic (social, economic, and environmental) value and maximize benefits to people and society (Table 1). Applying these methodologies provides a direct route and useful evidence for including and considering health and equity in health and non-health sector decision-making, prioritization and investment processes, thus contributing to the “Health in all Policies” agenda and reducing health inequity (4).

**Table 1:** Key steps of the Health Impact Assessment (HIA) and Social Return on Investment (SROI) methodologies.

**Health impact assessment**
Step 1 Screening: Determining whether a HIA is warranted and whether it would be valuable in the decision-making process. This includes understanding what is already known and identifying gaps. Step 2 Scoping: Using the results of the screening stage to decide on the scope of the assessment, including data sources to use, affected groups/populations to cover, identification of stakeholders and methods of engagement. Step 3 Appraisal of evidence: Undertaking the defined methodology and triangulating both qualitative and quantitative evidence. Step 4 Reporting and recommendations: Produce a written report and disseminate what was learned to the community in question. Step 5 Review and reflection including monitoring and evaluation: Monitor implemented recommendations to see if they are working as expected and evaluate the process itself.
**Social return on investment**
Step 1 Establishing scope and identifying stakeholders: Setting clear boundaries about what the SROI will cover, who will be involved in the process and how. Step 2 Mapping outcomes: Engaging qualitatively with stakeholders to develop an impact map or theory of change to show the relationship between inputs, outputs and outcomes. Step 3 Evidencing outcomes and giving them a quantifiable value: Finding data to show whether outcomes have occurred and placing a value on them. Step 4 Establishing impact: Undertake process to account for change which would have happened anyway or are a result of other factors. Step 5 Calculating the SROI: Adding up all of the benefits, subtracting any negatives and comparing the result to the investment. Step 6 Reporting, using and embedding: Sharing findings with stakeholders.

HIA is a well-known methodology, commonly defined as “a combination of procedures, methods, and tools by which a policy, intervention or service may be judged as to its potential effects on the health of a population, and the distribution of those effects within a population” (5). Developed as a flexible and scalable tool, HIA allows health and well-being to be considered in all policy arenas as a method of implementing “Health in all Policies” approaches, and has the power to influence the decision-making process by promoting cross-sector collaboration (6). As practiced in Wales and applied by the Wales Health Impact Assessment Support Unit (WHIASU), HIA is a systematic, equity focused and participatory process which views health in its holistic definition (7). It also considers all possible impacts through a wider determinant lens, including health behaviors, social and community networks, living and working conditions, and general socioeconomic, cultural, and environmental conditions which can influence individual choice and population behavior (8). Broad approach HIA (which is based on mixed methods of evidence collection including health intelligence and other data and research, qualitative and quantitative evidence) focuses on the implications for the community, population and health equity (by focusing on those affected and how any impacts may be distributed), and directly involves stakeholders who have an interest in, or are affected by the public policy, plan, intervention or service under assessment. Thus, HIA has a method and evidence driven, inclusive participatory nature that incorporates high levels of stakeholder engagement throughout the process and accounts for multiple wider impacts, as well as health, well-being and (in)equality implications. This includes a stakeholder analysis to identify specific and appropriate levels of engagement taking into account the nature of assessment and context of the proposal to be assessed. Outcomes identified by individuals traditionally through methods of participatory workshops, interviews, and focus groups are presented collectively in a written format to relevant stakeholders (6, 9) to inform and aid decision making. As applied in Wales, HIA follows a “salutogenic approach” that takes into account the wider social and health and well-being and what contributes to well-being and being well, rather than solely focusing on the impact of disease on health and what makes individuals and populations sick. In addition, HIA in Wales does not only focus on the negative impacts of some purely tight approach and risk-focused HIAs or health integrated Environmental Impact Assessments (EIA), but also takes into account the potential positive impacts and any opportunities for health improvement.

Although HIA is a recognized robust methodology, existing evidence suggests that impact assessments could potentially benefit from methods to quantify the effect of social and economic outcomes (10, 11). Particularly with the introduction of the Public Services (Social Value) Act 2012 in the United Kingdom (12), a wide range of sectors are considering how to measure social value through economic analysis in order to aid and inform decisions. For example, capturing, and understanding social value (such as the value created to individuals, families, communities and the environment) in relation to the built environment and infrastructure development (13).

New economic methodologies have been developed to better understand, evaluate and value the wider societal impact of different interventions and policies, thus capturing and measuring their “social value” (14). SROI is defined as an economic method, which accounts for the broader concept of social value by incorporating social, environmental and economic costs and benefits in the valuation. Unlike traditional economics methodologies, such as cost-effectiveness and cost-benefit analyses, SROI is looking also at non-financial impacts that add real value to people's lives, to communities and society, and to the wider economic and environmental setting. Through a stakeholder engagement, the SROI defines outcomes, and allows a monetary value to be placed on the non-financial returns on investment by applying proxy values (15). This approach provides a holistic framework, reflecting the wider determinants of health (and sometimes equity) by valuing outcomes, which are not measured by the traditional financial value for money approaches (Table 1). This is particularly relevant for public health policies and interventions, which usually have multiple “soft” and difficult to quantify impacts, such as improving or promoting health, well-being and equity of population groups and communities, as well as bringing additional benefits to their living, social, or working environment (16, 17).

The methodological processes of HIA and SROI indicate synergies and cross-over in their approaches. Combining the two methodologies, running them alongside one another, or using HIA as a platform to build on, could have an added value to both and provide useful information for decision-makers. The Institute of Environmental Management and Assessment (IEMA) notes how the “social value” approach used within SROI can be applied to a range of health determinants within health and well-being checklists used within HIAs (18). Assigning a monetary value to these impacts can give greater clarity to the concluding report and possible application and impact of a HIA. And vice-versa, HIA can provide a well-structured robust platform for the SROI stakeholder engagement, participation, and gathering evidence, also ensuring the balance in use and interpretation of difference types of evidence.

Vice-versa, stakeholder engagement methodologies outlined in the SROI process have been described as vague in their approach, particularly in relation to stakeholder participation, and have been claimed to prioritize stakeholder evidence over other types (19, 20). In addition, HIA can complement the SROI method in understanding how and why impacts occur, which can help decide which outcomes would be more important or useful to valuate (21).

The World Health Organization (WHO) Collaborating Center (CC) on Investment for Health and Well-being at Public Health Wales and WHIASU have started exploring the synergies and potential joint application of HIA and SROI to assess and measure impact and value of public policies, interventions, services, or planning. Within this paper, we explore and discuss how HIA and SROI methodologies could complement each other to capture and account for the multiple health and well-being, social, economic, equity and environmental impacts, and the holistic value of an assessed intervention or policy. We investigate how HIA and SROI approaches have been used in combination previously, aim to understand how the two methodologies could be linked in practice and discuss the added value of doing so.

## Materials and Methods

A scoping review of existing academic and gray literature was undertaken to identify case studies where HIA and SROI methodologies have been used in tandem previously to understand the potential impact of a policy, service, or intervention. The search terms used to search within peer reviewed databases (PubMed and ProQuest) were “Health Impact Assessment” or “Impact Assessment,” and “Social Return on Investment” or “Social Value.” The gray literature was also searched using the same search terms to identify any existing evidence. Evidence was identified via review of titles, abstracts, or executive summaries, found by searching on Google Scholar and organizational websites (WHIASU, World Health Organization, Social Value UK).

For both the academic and gray literature, we searched for evidence published between January 1996 and April 2019. This timeframe was chosen as the first SROI study was published in 1996 so it was agreed no published literature would be found prior to this date. Snowballing techniques were also used to identify and capture additional studies, alongside communications with SROI and HIA practitioners to provide any further examples that they were aware of. Two researchers independently conducted the search and initial screening of findings. Final inclusion of studies was based on strict inclusion and exclusion criteria. Evidence was only included if it was published in the timeframe identified above, included reference to both HIA and SROI, and was published in the English language. A data extraction table was used to capture study information and informative findings for the purpose of this study.

Finally, semi-structured qualitative interviews were undertaken with key international experts from a range of regulatory and legislative contexts (i.e., United States, Australia, and the United Kingdom) to gain a deeper understanding of their past experiences and expertise of both HIA and SROI methodologies. For the purpose of this research, approval from an Ethics Committee was not required as per guidance from the NHS Health Research Association ethics decision tool. This research posed no potential risk to the individuals participating, and no participant identifiable data was collected from respondents. Individuals who had knowledge of HIA and/or SROI methodologies were identified from the small number of case studies identified in the scoping review, and existing networks. These interviews explored past experiences to gain insight into current thinking and the potential for future developments of the methodologies. Potential participants were approached with an invitation to interview via email, alongside an information sheet including a description of the HIA and SROI methodologies. Informed consent was collected by the interviewers ahead of the interviews. Interviews were undertaken either face-to-face or via telephone, depending on the location of the interviewee. All interviews were digitally recorded and transcribed by a professional transcription company. The interviews followed a semi-structured approach, which allowed participants to describe their experiences and expertise at length, but participants were gently guided to discuss areas of particular interest. The questions within the interview schedule were guided by and developed through initial exploratory work reviewing the main guidance documents for the two methods. Developed by WHIASU, “*Health Impact Assessment. A Practical Guide*” (9) was used as a guidance resource to fully understand the HIA process and established steps for undertaking a formal HIA. For SROI, “*A guide to Social Return on Investment*” (15) outlines the SROI process in stages and allowed the study team to think through and discuss potential overlaps between the two methodologies, and also appreciate the potential benefits and weaknesses. Topics covered by the interview questions included the following: past experience of HIA and SROI, benefits of both HIA and SROI, thoughts on how either methodology could be changed or improved, potential overlaps between the two methodologies, whether they had used the two methods before in tandem, what the added value would be of running the two in tandem, and whether there would be any potential negative effects of doing so. A thematic analysis was undertaken on the interview data collected.

## Results

### Scoping Review

The scoping review identified two published reports on scenarios where HIA and SROI have both been used to assess the same intervention or policy. These findings were identified from within the gray literature, with no findings being detected through the academic literature search. The identified case studies are outlined in Table 2 and highlight the potential use of the two methodologies to evaluate and present the wider impacts and social value.

**Table 2:** Case studies identified within the scoping review.

**Health disability sport partnership**
Funded by Sports Wales in 2015, Disability Sport Wales (DSW) in partnership with Betsi Cadwaladr University Health Board (BCUHB) undertook a standalone retrospective HIA which was completed as part of a mid-term evaluation of the Health Disability Sport Partnership (22). The aim of the partnership was to improve the health and well-being of disabled people by up-skilling health professionals and supporting them to signpost disabled people toward physical activity. Supported by the Wales Health Impact Assessment Support Unit (WHIASU), a community profile was developed of people with disabilities and physical activity levels in the area. A stakeholder group was established and the impact of the intervention was assessed, not only for participating individuals, but also for a wide range of stakeholders such as family and health professionals. An action plan was developed at the end of the HIA process. In addition to the HIA process, an evaluative SROI was undertaken on the intervention in 2016 (23) with the aim of capturing the social value created by the partnership. The outcomes identified in the previous HIA process were used in the SROI, and were developed by additional stakeholder interviews. The Housing Associations' Charitable Trust (HACT) Social Value Bank (24) was then used to assign a monetary value to outcomes where possible. Costs provided by the Health Board were used to calculate the inputs to the intervention and enable the SROI ratio to be calculated. The final SROI report concluded that for every £1 invested, £124 of social value was created.
**“Secure Warm Modern” homes in Nottingham: Decent Homes Impact Study**
In their evaluation of the Decent Homes Impact Study (25), Nottingham City Homes carried out both a HIA and SROI. The initiative was introduced to improve the homes of social housing tenants by addressing a backlog of repairs in local authority housing. In partnership with Nottingham Trent University and Nottingham City National Health Service (NHS), a HIA was undertaken to understand the wider health impact of the Decent Homes service as part of the social inequalities in health section of the wider service. An SROI was also carried out on a case study area in receipt of the service to create a theory of change to measure the social, environmental and economic outcomes through stakeholder engagement. The final report indicated that for every £1 invested, £4.76 of social value is generated.

### Semi-structured Interviews

Due to available resources and timeframes allocated to this stage of the study, 13 international HIA Impact Assessment and SROI experts were invited to participate in an interview. Nine responded to the invite and were interviewed in October 2019. The number of interviews achieved satisfied the needs of this initial exploratory study with responses gained from a wide range of individuals who were keen to engage in this work. Of those nine, three individuals were experienced in the use of HIA, two had experience of being involved in one previous HIA, with the remainder having no past experience. With regards to SROI, five individuals reported past experience of using SROI methodology, one had undertaken formal training in SROI but had no experience to date of putting the training into practice, and three individuals had no experience of SROI. Two individuals had experience of using both methods previously. The mean length of interview was 25.5 min.

### Strengths of HIA and SROI as Standalone Methodologies

When asked for thoughts on the benefits of undertaking a HIA, just over half of the respondents (*n* = 5) agreed that the HIA process is well-structured using prescriptive method, with two participants reporting that they felt HIA is a methodology that considers health and well-being in its broadest context. Just under half of the respondents (*n* = 4) recognized the importance of involving stakeholders in the HIA process and the tangible benefits from involving different parties (individuals and organizations) who may be directly or indirectly affected by a proposal or plan. It was thought that enabling participation through robust methods such as workshops and interviews allowed stakeholders to get their voices heard. In addition, several respondents highlighted the importance of both the screening and scoping stages to enable planning and engagement with a wider range of individuals, which provides a higher validity to the methods undertaken. Other benefits of the HIA approach which were highlighted by interviewees was the strong focus on health inequalities, using a clear set of principles and following a process which is scalable and flexible, and can be used both prospectively and retrospectively.

In response to identifying the benefits of undertaking an SROI, all respondents stated the added value of the importance of monetizing outcomes to help show what difference the service or intervention has made. One respondent noted that this can be particularly useful in relation to funding decisions, and in the field of public health:

*So it is a really good way of quantifying things that you wouldn't think are quantifiable…put a cost to things that actually don't bring money back in but just have that value in the future for people's health and the wider economy*.

Over half of the respondents (*n* = 5) noted the positive impact of recognizing and seeking to quantify the triple bottom line (social, economic, and environmental) of outcomes and impacts:

*I think it's a really useful way of sort of combining multiple types of outcomes in a sort of consistent framework. So anything from environmental outcomes, to health and local economic outcomes, you can kind of put them all in the mix*.

*It places health and well-being in its broadest sense at the heart of the considerations which I do not feel conventional health economic analysis does. It also places those who are directly affected by or involved in the intervention at the heart of the analysis*.

Another identified benefit of the SROI method which emerged as a theme, was the strong emphasis on stakeholder engagement. It was noted that this approach enables an inclusivity by combining perspectives of different people (and organizations), not only those who are participating in an intervention but also wider stakeholders, such as family and friends:

*It's obtaining the participant voice and the value they place on an outcome*.

Additional benefits of SROI raised by respondents were also the process of impact mapping through which all inputs and outcomes are identified, and also accounting for different factors which would have happening anyway to avoid overstating the impact.

All strengths and weaknesses of the methodologies as reported by respondents are outlined in Table 3.

**Table 3:** Identified strengths and limitations of HIA and SROI.

	**Strengths**	**Limitations**
HIA	• Well-structured and prescriptive method. • Considers health and well-being in its broadest context. • Stakeholder involvement and participatory in nature. • Screening and scoping stages to enable planning and engagement with a wide range of individuals. • Focus on health inequalities. • Can be used prospectively, concurrently and retrospectively. • Scalable and flexible.	• No economic quantification of outcomes. • Screening and monitoring/evaluation stages not undertaken throughly in some HIAs. • Room for methodological development and evolution. • Some HIAs can be narrowly focused i.e., assess only environmental determinants as part of wider impact assessment processes.
SROI	• Allows for monetization of social, environmental and economic outcomes. • Stakeholder engagement and participatory in nature. • Can be used prospectively and retrospectively.	• Valuation process needs to be improved to reduce subjectivity. • Process of stakeholder engagement to identify outcomes needs to be improved to increase quality. • No use of checklists to ensure clear process

### Similarities Between HIA and SROI Methodologies

By outlining the benefits of both methodologies, it is possible to identify similarities and synergies between them. All interviewees recognized the common approach in both methods, looking at the holistic value and the wider determinants of health and equity through stakeholder engagement and involvement throughout the process. It was also noted that both processes: acknowledge unintended consequences and impacts; increase the accuracy and validity of their results through being inclusive of the relevant stakeholders and related outcomes; use methods of triangulation on both qualitative and quantitative data; can be carried out prospectively or retrospectively; and follow social values and principles, such as equity, diversity, inclusion, and transparency. A number of similarities were identified between stages of both the HIA process and the SROI process. For example, both begin with a scoping stage, carry out similar reporting procedures, and monitoring and quality assurance.

### Potential for Adding Value to the HIA and SROI Methodological Processes

Interviewees were also questioned about their thoughts on what elements could add value to either of the two processes to help us explore how the two processes could potentially complement one another (see HIA and SROI stages in Table 1). With regards to HIA, respondents noted the following: the Screening and Monitoring stages could be used to better potential as sometimes the Stages can lack emphasis and commitment by those undertaking the HIA; further methodological developments are needed to better engage with vulnerable stakeholders identified in the scoping stage, but not typically engaged with later in the process, to collect outcomes data; and outcomes are not currently quantified in an economic sense (monetized) to capture their value.

With regards to the SROI process, a theme emerged regarding the heavy importance placed on evidence collected from stakeholders over other types of evidence. In addition, it was suggested that it needs to be made clearer at the reporting stage of the process that the monetary value produced is only a proxy value, as opposed to an actual financial figure which needs to be communicated in an appropriate way to the target audience.

### Benefits and Disadvantages of Undertaking HIA and SROI on the Same Intervention

A number of benefits were highlighted by eight out of the nine interviewees regarding the potential benefits of running both methodologies on the same intervention, service or policy (Table 4).

**Table 4:** Identified benefits and disadvantages of undertaking a HIA and SROI on the same intervention.

**Benefits**	**Disadvantages**
• Using both processes allows the illustration of the well-rounded impact of the service, intervention or policy. • The use of impact mapping in the SROI process would benefit the HIA process to help identify the outcomes in addition to the methods already in place in HIA methodology. • Adding a monetary value to outcomes through the SROI process can help to build a more compelling case for health and well-being • Undertaking an SROI after completing a HIA can help to focus on the necessary stakeholders to be involved in the SROI process. • Using the HIA checklists within SROI can help to address focus on inequalities and vulnerable groups.	• Running both processes may cause confusion, so would need to be communicated effectively to understand the added value. • Need to ensure the focus on health and equity in HIA isn't lost through a change in focus to the monetization of outcomes.

When questioned how this would work in practice, the main emerging theme was to potentially use the HIA approach for initial exploration of impact when undertaking a prospective evaluation, and to use HIA for the scoping stages to identify impact, then move toward then use SROI to quantify the outcomes previously identified in the HIA:

*So if I hadn't done the HIA and just come straight across to social return on investment, the first step I would've done would have essentially still been a HIA because you still had to collect all the outcomes from your wider stakeholders to be able to set your outcomes for you to be able to do the social return on investment. So, actually, I think it, it wouldn't, to me, have made sense to do a social return on investment without having done the HIA first*.

However, it is important to note that it very much depends on the service or intervention being assessed as to what methodology would be the most beneficial. For example, one interviewee commented that undertaking SROI at a strategic policy level may be more challenging than undertaking one on a local intervention. By undertaking an SROI after a HIA, it was suggested that it presents the opportunity to provide stakeholders with additional background from the HIA process to help focus their approach to quantifying the value.

As reported in Box 3, the theme of monetizing the outcomes identified through the HIA process by using SROI emerged strongly:

*It is having a quantifiable element given many large-scale infrastructure developments/service reconfigurations require an economic element to business cases*.

In addition, some interviewees commented how the two methodologies could learn from each other. For example, a theme emerged that the HIA process could be more explicit when stating why particular stakeholders are included or excluded from the engagement processes. An additional comment was to think about incorporating the impact more explicitly within HIA participatory workshops to focus on short, medium and long-term outcomes. With regards to improving the SROI methodology, several interviewees suggested that the methodology could be enhanced if elements of the HIA process were adopted, such as the use of the wider determinants checklists.

## Discussion

Overall, this study has identified very limited evidence of applying both HIA and SROI methodologies on the same policies, services or interventions. The two case studies found and acknowledged in this scoping review have provided some context of how they have been used together previously to aid the evaluation and future development of services (22, 23, 25). These examples may reflect that the two methodologies discussed in this paper can be applied together on interventions or services, as opposed to assessing a wide range of impact and social value of strategic initiatives and policies. In addition, the lack of academic literature found in this scoping review is reflective of other reviews which note the little uptake of SROI methodology by academics (16, 20). This could potentially be due to lack of resources to use SROI methodology, or a lack of awareness or understanding by practitioners and policy makers of the methodology. This could be determined through further research.

By obtaining viewpoints from both experts in HIA and SROI, we were able to identify themes related to how the two methodologies could be used together to develop and potentially advance approaches to capturing impact on health and well-being, and social value. Results have indicated that both methodological processes involve a number of similar elements. Both methods are underpinned by a social or holistic model of health, recognizing that health outcomes are shaped by wider social, economic and environmental factors, and follow similar principles such as stakeholder participation and transparency (9, 15). The two methods strongly understand that health means different things to different people, capturing this through engagement with multiple stakeholders to portray both intended and unintended outcomes. This links with existing literature that notes the relationship between depth of involvement of stakeholders in a service or intervention and the likelihood of good social value outcomes (13). Finally, it was noted that both methods can be used prospectively, concurrently, or retrospectively.

**Figure 1:**
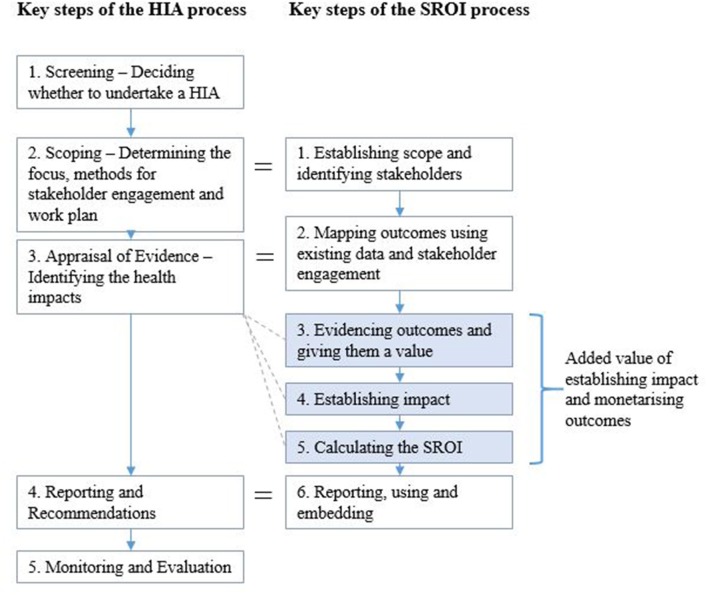
Similarities and gaps between the HIA and SROI process.

Existing evidence regarding both HIA and SROI methodologies individually states that they can be used in their own right to inform and support effective decision-making, and to drive dialogue and decisions to be outcome-focused, taking into account the wider determinants of health and health equity (15, 26–29). However, this research has highlighted some fundamental differences between the methods, which illustrate how they could be used to complement each other and potentially fit into existing processes (Figure 1). For example, although heavily promoted within SROI, engagement methods used could be strengthened by replicating approaches taken within the HIA approach. For example by using the health and well-being and population checklists (9) which list a range of health and well-being determinants and outcomes and population groups who could be potentially affected. This could ensure a wide range of potential outcomes and indicators are covered, including vulnerable groups, inequities and inequalities. SROI was also noted to potentially privilege stakeholder perspectives over other types of evidence (20) which could be avoided by triangulation used within HIAs to consider and compare different available evidence.

A major benefit of the SROI process which currently does not exist in HIA has been identified within this research, as the process of placing a monetary value on outcomes not traditionally quantified (Figure 1). There is the potential for adding this into the Appraisal stage of a HIA (Figure 1). However, if this is the main factor which results in HIA and SROI being run on the same intervention, it is important that subjectivity is accounted for within the valuation process, including remaining cautious around overstating the value of the SROI ratio and how it is used so that the focus on health equity highlighted through the HIA process isn't displaced.

Although this paper has taken the first steps to understand the relationship between SROI and HIA methodologies, there is major scope for future research to be undertaken to develop the concept of applying the two methodologies in combination to accurately measure and report the health and well-being impact, as well as the social value of services, polices or interventions. Due to the limited published evidence and literature in this space, further research and learning from case studies is needed, in order to better comprehend the advantages and disadvantages, and to further develop both SROI and HIA methodologies. In addition, the relatively small number of interviews undertaken in this study is acknowledged and reflects the limited practice of utilizing both methods together. Extra benefit would be created by capturing the views of decision-makers who would be using the results to understand if they would add value and how are they going to apply them.

Evidence suggests there is current limited knowledge about how to embed social value in the development of strategies or services in some sectors (30). The UK Green Business Council (UK GBC) note that although HIA is used within the sector, there is not yet a common methodology for measuring social value in real estate, or an industry wide framework to provide consistency (13). Further methodological developments in both HIA and SROI could promote a framework or process to capture this, however this would require further investigation.

## Conclusion

Despite the increasing use of HIA and SROI methodologies at an international level, very little previous research has been undertaken to investigate how these approaches complement each other to capture and account for the health impacts and the social value of policies, interventions, and services. This paper demonstrates that there are some clear synergies between the two methodologies within their well-established processes, principles and reliance upon stakeholder engagement to identify the real outcomes, both experienced and predicted. Our research also indicates the added value of applying both processes (in tandem or sequentially) to help measure health impact and social value due to the subtle differences, for example the checklists used within the HIA process and the monetization of outcomes in the SROI methodology. Although this review identified the two methodologies had only been applied twice previously on the same intervention, the conducted interviews acknowledged scope to continue to develop this work further. This understanding and building evidence could inform and enable decision-makers to incorporate health impact and social value in developments and initiatives across sectors, on a national and local level. This is going to be explored further by the WHO CC and WHIASU, in collaboration with partners nationally and internationally.

## Data Availability Statement

The qualitative datasets for this article will not be made publicly available as consent was not gained from interview respondents to share transcripts external to the study team. Requests to access these datasets should be directed to KA (kathryn.ashton@yahoo.co.uk).

## Author Contributions

KA and LG developed and designed the research. KA undertook the scoping review. KA and LP-W undertook the qualitative data collection. KA analyzed the data and drafted the manuscript. All authors edited and approved the final manuscript.

### Conflict of Interest

The authors declare that the research was conducted in the absence of any commercial or financial relationships that could be construed as a potential conflict of interest.

## References

[1] Cole BL Fielding JE. Health impact assessment: a tool to help policy makers understand health beyond health care. Annu Rev Public Health. (2007) 28:393–412. 10.1146/annurev.publhealth.28.083006.131942 17173539

[2] Lock K. Health impact assessment. BMJ. (2000) 320:1395–8. 10.1136/bmj.320.7246.1395 10818037 PMC1118057

[3] World Health Organization Health Impact Assessment (HIA). (2019). Available online at: https://www.who.int/hia/en/ (accessed November 12, 2019).

[4] The American Public Health Association. Promoting Health Impact Assessment to Achieve Health in All Policies. (2012). Available online at: https://www.apha.org/policies-and-advocacy/public-health-policy-statements/policy-database/2014/07/11/16/51/promoting-health-impact-assessment-to-achieve-health-in-all-policies (accessed November 12, 2019).

[5] European Centre for Health Policy Health Impact Assessment. Main concepts and suggested approach. Gothenburg Consensus Paper (1999). Available online at: http://www.impactsante.ch/pdf/HIA_Gothenburg_consensus_paper_1999 (accessed November 12, 2019).

[6] Collins J Koplan JP. Health impact assessment. A step toward health in all policies. JAMA. (2009) 302:315–7. 10.1001/jama.2009.1050 19602691

[7] World Health Organization Constitution of the World Health Organization. (1948). Available online at: http://apps.who.int/gb/bd/PDF/bd47/EN/constitution-en.pdf (accessed November 12, 2019).

[8] Whitehead M Dahlgreen G Concepts and Principles for Tackling Social Inequalities in Health: Levelling Up Part 1. (2006). Available online at: http://www.enothe.eu/cop/docs/concepts_and_principles.pdf (accessed November 12, 2019).

[9] Wales Health Impact Assessment Unit Health Impact Assessment. A Practical Guide. (2012). Available online at: https://whiasu.publichealthnetwork.cymru/files/1415/0710/5107/HIA_Tool_Kit_V2_WEB.pdf (accessed November 12, 2019)

[10] Veerman JL Barendregt JJ Mackenbach JP. Quantitative health impact assessment: current practice and future directions. J Epidemiol Community Health. (2005) 59:361–70. 10.1136/jech.2004.026039 15831683 PMC1733071

[11] Treasury HM The Green Book. Central Government Guidance on Appraisal and Evaluation. (2018). Available online at: https://assets.publishing.service.gov.uk/government/uploads/system/uploads/attachment_data/file/685903/The_Green_Book.pdf (accessed November 12, 2019).

[12] UK Government Public Services (Social Value) Act. (2012). Available online at: http://www.legislation.gov.uk/ukpga/2012/3/enacted (accessed November 12, 2019).

[13] UK Green Business Council Social Value in New Development: An Introductory Guide for Local Authorities and Development Teams. (2018). Available online at: https://www.ukgbc.org/wp-content/uploads/2018/03/Social-Value.pdf (accessed November 12, 2019).

[14] Kemm J Health impact assessment and health in all policies. In: Stahl T, Wismar M, Ollila E, Lahtinen E and Leppo K, editors. Health in All Policies. Prospects and potentials. Finland: Ministry of Social Affairs and Health (2006). p. 189–207.

[15] Social Value UK A Guide to Social Return on Investment. Available online at: http://www.socialvalueuk.org/app/uploads/2016/03/The%20Guide%20to%20Social%20Return%20on%20Investment%202015.pdf (accessed November 12, 2019).

[16] Banke-Thomas AO Madaj B Charles A van den Broek N. Social Return on Investment (SROI) methodology to account for value for money of public health interventions: a systematic review. BMC Public Health. (2015) 15:582. 10.1186/s12889-015-1935-7 26099274 PMC4477315

[17] Masters R Anwar E Collins B Cookson R Capewell S. Return on investment of public health interventions: a systematic review. J Epidemiol Commun Health. (2017) 71:1–8. 10.1136/jech-2016-208141 28356325 PMC5537512

[18] Institute of Environmental Management and Assessment (IEMA) EIA Quality Mark Article. Health Impact Assessment (HIA) and Social Value. (2017). Available online at: https://www.iema.net/assets/uploads/EIA%20Articles/Turley%20Health%20Impact%20Assessment%20(HIA)%20and%20Social%20Value.pdf (accessed November 12, 2019).

[19] Fujiwara D The Seven Principle Problems of SROI. (2015). Available online at: http://www.socialvalueuk.org/app/uploads/2016/03/The%20Seven%20Principle%20Problems%20with%20SROI_Daniel%20Fujiwara.pdf (accessed November 12, 2019).

[20] Hutchinson CL Berndt A Forsythe D Gilbert-Hunt S George S Ratcliffe J. Valuing the impact of health and social care programs using social return on investment analysis: how have academics advanced the methodology? A systematic review. BMJ Open. (2019) 9:e029789. 10.1136/bmjopen-2019-029789 31446413 PMC6720245

[21] Arvidson M Lyon F McKay S Moro D The ambitions challenges of SROI. Third Sector Research Centre Working Paper 49. (2010). Available online at: http://bigpushforward.net/wp-content/uploads/2011/09/the_ambitions_and_challenges_of_sroi.pdf (accessed November 12, 2019).

[22] Chin C Health Disability Sport Partnership. Health Impact Assessment. (2015). Available online at: http://www.wales.nhs.uk/sites3/Documents/522/Health%20Impact%20Assessment-%20Health%20Disability%20Sport%20Partnership.pdf (accessed November 12, 2019).

[23] Chin C Health Disability Sport Partnership: A Social Return on Investment Analysis. (2016). Available online at: https://whiasu.publichealthnetwork.cymru/files/6715/0210/7630/The_Health_Disability_Sport_Partnership_-_A_Social_Return_on_Investment_Analysis_FINAL.pdf (accessed November 12, 2019).

[24] Housing Associations' Charitable Trust Social Value Bank. (2015). Available online at: https://www.hact.org.uk/social-value-bank (accessed November 12, 2019).

[25] Nottingham City Homes The effects of ‘Secure Warm Modern' homes in Nottingham: Decent Homes Impact Study. (2016). Available online at: https://www.nottinghamcityhomes.org.uk/repairs-and-improvements/improving-your-home/the-impact-of-improving-your-home/ (accessed November 12, 2019).

[26] Dreaves HA. How Health Impact Assessments (HIAs)help us to select the public health policies most likely to maximise health gain, on the basis of best public health science. AIMS Public Health. (2016) 3:325–41. 10.3934/publichealth.2016.2.235 29546158 PMC5690350

[27] Heller J Marjory LG Yuen TK Gould S Benkhalti Jandu M Bourcier E Choi T. Advancing efforts to achieve health equity: equity metrics for health impact assessment practice. Int J Environ Res Public Health. (2014) 11:11054–64. 10.3390/ijerph111111054 25347193 PMC4245599

[28] Dyakova M Hamelmann C Bellis MA Besnier E Grey CNB Ashton K Schwappach A Clar C (2017). Investment for health and well-being: a review of the social return on investment from public health policies to support implementing the Sustainable Development Goals by building on Health 2020. Health Evidence Network synthesis report 51 (2017). Available online at: http://www.euro.who.int/en/publications/abstracts/investment-for-health-and-well-being-a-review-of-the-social-return-on-investment-from-public-health-policies-to-support-implementing-the-sustainable-development-goals-by-building-on-health-2020-2017 (accessed November 12, 2019). 28956895

[29] Association of Project Management Social Return on Investment (SROI): A Powerful Tool for the Realisation of Benefits. (2016). Available online at: https://www.apm.org.uk/media/2218/sroi-report-2016-web-final.pdf (accessed November 12, 2019).

[30] Thurairajah N Muchena J Xiao H Challenges to embedding Social Value Act 2012 in the strategic and operational processes of public sector construction projects. In: Proceedings of the 25^th^ Annual ARCOM Conference, Leeds, 2–4. (2019). p. 71–80.

